# Simulated Gastrointestinal Digestion and In Vitro Fecal Fermentation of Purified *Pyracantha fortuneana* (Maxim.) Li Fruit Pectin

**DOI:** 10.3390/foods14091529

**Published:** 2025-04-27

**Authors:** Qingrui Xu, Yiyi Lv, Xiaohui Yuan, Guichun Huang, Zhongxia Guo, Jiana Tan, Shuyi Qiu, Xiaodan Wang, Chaoyang Wei

**Affiliations:** 1 Key Laboratory of Fermentation Engineering and Biological Pharmacy of Guizhou Province, School of Liquor and Food Engineering, Guizhou University, Guiyang 550025, China; 2 Key Laboratory of Plant Resource Conservation and Germplasm Innovation in Mountainous Region (Ministry of Education), Institute of Agro-Bioengineering, College of Life Sciences, Guizhou University, Guiyang 550025, China

**Keywords:** *Pyracantha fortuneana*, pectin, gut microbiota, simulated in vitro digestion, fecal fermentation

## Abstract

*Pyracantha fortuneana*, an underutilized wild plant, has been found to have a high nutritional value. This study used simulated digestion and fecal fermentation models to investigate the digestive properties of the purified acidic pectin polysaccharide of *Pyracantha fortuneana* and its impact on the gut microbiota and metabolites. *Pyracantha fortuneana* polysaccharide (PFP) is mainly composed of rhamnose (Rha), galacturonic acid (GalA), glucose (Glc), galactose (Gal), and arabinose (Ara), with a molecular weight (Mw) of 851.25 kDa. Following simulated digestion, the Mw of PFP remained consistent. The reduced sugar content showed minimal change, suggesting that PFP exhibits resistance to gastrointestinal digestion and can effectively reach the colon. Following fecal fermentation, the molecular weight, monosaccharide, and carbohydrate contents of PFP decreased, while the short-chain fatty acid content increased. This suggests that PFP is susceptible to degradation by microorganisms and can be metabolized into acetic acid and *n*-butyric acid, contributing to the regulation of intestinal health. Meanwhile, PFP promotes the reproduction of beneficial bacteria such as *Bacteroides*, *Dialister*, and *Dysgonomonas*, inhibits the growth of harmful bacteria like *Proteus*, and generates metabolites such as thiamine, leonuriside A, oxoadipic acid, S-hydroxymethylglutathione, and isonicotinic acid, which exert beneficial effects on human health. These results indicate that PFP has great potential in regulating the gut microbiota and generating beneficial metabolites to promote intestinal functional health and can be used as a prebiotic to prevent diseases by improving intestinal health.

## 1. Introduction

*Pyracantha fortuneana* (Maxim.) Li, a plant species of Maloideae within the *Pyracantha Roemer* genus, is commonly found in the southern and northwest regions of China and is frequently used for the treatment of indigestion [1]. The fruit of this plant is rich in bioactive substances such as polysaccharides, polyphenols (especially anthocyanins and flavonoids), and dietary fiber, thus making it a valuable natural resource [2]. Among these components, polysaccharides stand out as a key bioactive compound, exhibiting various beneficial effects, including enhanced immune function and antioxidant properties [3]. Positive correlations were observed between the administration of *P. fortuneana* polysaccharide and the enhancement of immune function and antioxidant activity in mice [4]. Furthermore, polysaccharides extracted from *P. fortuneana* exhibited significant cytotoxicity in Skov3 cells by activating intracellular reactive oxygen species (ROS) production, resulting in mitochondrial disruption and DNA damage, ultimately leading to apoptosis in ovarian cancer cells [5]. A previous study employed six methods to extract and prepare polysaccharides from *P. fortuneana*, thereby elucidating their physicochemical properties, structural characteristics, and biological activities [6]. The polysaccharide extracted from *P. fortuneana* using acid extraction is identified as a plant pectin rich in galacturonic acid (GalA), which has sparked our interest.

**Figure 1:**
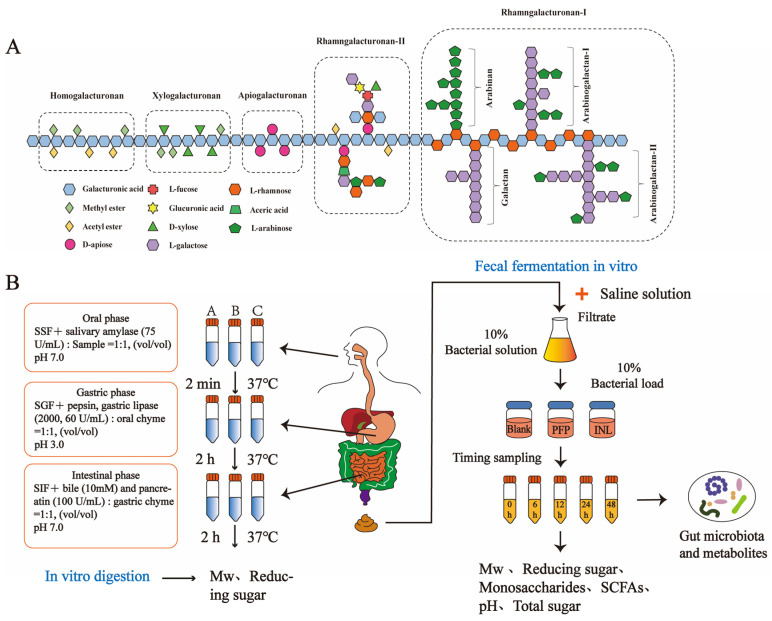
(**A**) Structural diagram of typical regions in pectin; (**B**) experimental scheme of PFP digestion and fermentation in vitro. Note: SSF (simulated saliva digestive fluid), SGF (simulated gastric digestive fluid), SIF (simulated intestinal digestive fluid), Mw (molecular weight), SCFAs (short-chain fatty acids). A, B, and C represent different experimental groups. The in vitro gastrointestinal digestion method shown in (**B**) follows the INFOGEST protocol.

Pectin is a complex anionic polysaccharide polymer rich in glucuronic acid, a biomolecule composed of GalA monosaccharides and some neutral monosaccharides by covalent cross-linking. Pectin can be classified into three main structural types based on differences in monosaccharide composition and glucoside bond: Homogalacturonan (HG), Rhamnogalacturonan I (RG-I), and Rhamnogalacturonan II (RG-II) [7]. Furthermore, two minor structural types, Xylogalacturonan (XGA) and Apiogalacturonan (AGA), are present in small quantities in certain fruits and lichenoid pectin [8]. Their specific structural domains are shown in Figure 1A. Currently, it has been revealed that the primary structure, degree of esterification, and molecular weight (Mw) of pectin significantly affect the nutritional function of the gut microbiota [9,10]. Pectin polysaccharides extracted from okra are rich in RG-I structures, whose content is positively correlated with the growth of anti-inflammatory microorganisms and the production of short-chain fatty acids (SCFAs) [11]. Meanwhile, pectin polysaccharides from *Lycium barbarum* and raspberry notably improved the intestinal ecological imbalance in mice suffering from inflammatory bowel disease (IBD) by enhancing immune organ parameters, repairing colon damage, reducing levels of TNF-α, IL-17, and IL-1β, alleviating oxidative stress, and boosting the overall production of SCFAs [12]. However, there is currently a lack of research reports on the prebiotic properties of pectic polysaccharides in *P. fortuneana*, such as their effects on the composition of the gut microbiota and SCFAs. This reveals that the structure of can be linked to alteration of the gut microbiota, which will shed more light on the prebiotic effects of the *Pyracantha fortuneana* polysaccharide (PFP).

This study examines the in vitro digestion and fermentation characteristics of the purified pectin polysaccharide PFP. The anti-digestion properties of PFP and its ability to reach the distal colon were evaluated using an in vitro simulated digestion model. Furthermore, the interaction between the pectin polysaccharide and intestinal health and its prebiotic potential was investigated using an in vitro fecal fermentation model. Assessment of intestinal fermentability, based on the production of SCFAs and alterations in the gut microbiota and metabolites, aimed at identifying beneficial flora regulated by the structural characteristics of PFP pectin for the study of functional foods.

## 2. Materials and Methods

### 2.1. Materials

In October 2022, *P. fortuneana* fruits were gathered from the local region (Weining County, Guizhou Province, China). The collected *P. fortuneana* fruits underwent drying and grinding and were subsequently passed through a 60-mesh sieve for preservation. Monosaccharide standards, including mannose (Man), ribose (Rib), rhamnose (Rha), glucuronic acid (GlcA), galacturonic acid (GalA), glucose (Glc), galactose (Gal), xylose (Xyl), arabinose (Ara), and fucose (Fuc) (a total of 10 types), were purchased from Beijing Solarbio Science & Technology Co., Ltd. (Beijing, China). The following enzymes from porcine sources, pepsin (250 U/mg) (EC: 232-628-3), trypsin (250 U/mg) (EC: 232-650-8), lipase (60 U/mg) (EC: 232-619-9), pancreatic enzymes (EC: 232-468-9), and bile, were also purchased from Beijing Solarbio Science & Technology Co., Ltd. (Beijing, China). The EC number refers to the Enzyme Commission nomenclature number. Dextran molecular weight standards ranging from 1 to 670 kDa were purchased from Sigma (St. Louis, MO, USA). All other chemicals and solvents used were of reagent grade and were purchased from The Sinopharm Group Co., Ltd. (Beijing, China).

### 2.2. Extraction and Purification of PFP

The extraction of pectin polysaccharides from *P. fortuneana* was carried out according to a previous study [6]. In other words, we extracted the powder of *P. fortuneana* using HCl (0.05 mol/L) for 2 h at 90 °C with a solid-to-liquid ratio of 1:30 (*w*/*w*). The supernatant was taken by centrifugation, and the extract was precipitated by adding anhydrous ethanol to a final ethanol concentration of 80% (*v*/*v*). The crude polysaccharide Ac-PFP of *P. fortuneana* was prepared by decolorization using an AB-8 macroporous adsorbent resin, deproteinization by the Sevag method after enzymolysis, and the extract was dialyzed and lyophilized in a 3500 kDa dialysis bag [13].

The crude Ac-PFP was dissolved in ultrapure water at a concentration of 20 mg/mL and then filtered through a 0.22 μm aqueous filter membrane. The filtrate was loaded onto a DEAE Sepharose Fast Flow anion exchange chromatography column (GE Healthcare, Little Chalfont, Buckinghamshire, UK) (24 × 400 mm) and eluted with ultrapure water and 0.1 M NaCl, 0.3 M NaCl, 0.5 M NaCl, and 2.0 M NaCl solutions at a flow rate of 4.8 mL/min using a peristaltic pump.

The 0.3 M NaCl elution fraction Ac-PFP-2 was collected according to the elution curve of Figure S2. Subsequently, the samples were concentrated and ultrafiltered using a Labscale TFF system (XX42LSS12, Millipore, Burlington, MA, USA) with a Pall 100 kDa ultrafiltration membrane package, and the fractions of different Mw were separated using a 0.3 M NaCl eluent. Monitoring was performed online using a high-performance gel permeation chromatography (HPGPC) system (LC-20AT, Shimadzu, Kyoto, Japan) until a single normally distributed peak appeared. The samples were desalted and collected. Collected samples were lyophilized to finally obtain a single component of purified PFP.

### 2.3. Physicochemical Characterization of PFP

#### 2.3.1. Composition of PFP

The total soluble sugar content of PFP was quantified using the phenol–sulfuric acid method and a mixed standard consisting of 80% GalA and 20% Glc [14,15]. The M-hydroxybiphenyl method was employed to measure the uronic acid content in PFP, using GalA as the standard material [16].

#### 2.3.2. Fourier Transform Infrared (FT-IR) and Ultraviolet Spectroscopy (UV) Analysis of PFP

The UV-1900i (Shimadzu Corporation, Kyoto, Japan), ranging from 190 to 600 nm, was used to detect the presence of proteins and nucleic acids. FT-IR spectroscopy (Nexus IS10 FTIR, Thermo Fisher Scientific, Waltham, MA, USA) was performed by mixing 1 mg of PFP with 100 mg of KBr and pressing it into thin slices, followed by transmission scanning in the range of 4000–400 cm^−1^.

#### 2.3.3. Determination of PFP Mw

The Mw was determined by an HPGPC system, which consisted of a high-performance liquid chromatograph (LC-20AT, Shimadzu, Kyoto, Japan) and two size-exclusion chromatography columns (Ultrahydrogel 2000 and Ultrahydrogel 250) (7.8 mm × 300 mm) (Waters Co., Milford, MA, USA) connected in series. To summarize, 3 mg of the sample were dissolved in 1 mL of the mobile phase, filtered through a 0.22 μm aqueous membrane, and then placed into a sample vial. A mobile phase of 0.2 M NaCl solution consisting of 0.03% NaN_3_ was used with a column temperature of 40 °C, an injection volume of 20 μL, a flow rate of 0.6 mL/min, and data collection for 50 min. LabSolutions v5.0 software was used for data acquisition and processing. The measured Mw was calibrated using a dextran molecular weight standard curve (1, 5, 12, 25, 50, 410, and 670 kDa), and the standard curve equation was y = −3.6168x + 45.328, R^2^ = 0.9915.

#### 2.3.4. Composition of Monosaccharide in PFP

The monosaccharide composition of PFP was prepared by pre-column derivatization with 1-phenyl-3-methyl-5-pyrazolinone (PMP) and identified by high-performance liquid chromatography (HPLC) [17]. The Waters 2695 HPLC system (Waters, Milford, MA, USA) was equipped with a PDA 2996 detector (Waters, Milford, MA, USA) and a Zorbax Eclipse XDB-C18 column (4.6 mm × 250 mm, 5 μm, Agilent, Santa Clara, CA, USA). Briefly, 3 mg of PFP were first mixed well with 1 mL of 4 M trifluoroacetic acid sealed in an ampoule and placed at 110 °C for 6 h of continuous hydrolysis. After acid hydrolysis, 0.2 mL of methanol were added to the hydrolysis product and dried with nitrogen, which was repeated three times to remove residual trifluoroacetic acid and dissolved in ultrapure water. Second, 450 μL of polysaccharide hydrolyzed samples or mixed standards were mixed with equal amounts of 0.3 M NaOH and 0.5 M PMP solution. This was bathed in water at 70 °C for 100 min, and the pH was adjusted to neutral with 0.3 M HCl. An equivalent volume of trichloromethane was added to remove residual PMP, after which the supernatant was filtered through a 0.22 μm membrane and stored for later use. Finally, the derivatized samples were studied by HPLC. The mobile phase was a mixture of phosphate buffer solution (0.05 M, pH = 6.8) and acetonitrile (15%, *v*/*v*). The flow rate and UV detection wavelength of the diode array detector were set at 1.0 mL/min and 250 nm, respectively, and the elution time was 45 min. Preparation of the mixed standard solution of monosaccharides involved dissolving each of the 10 monosaccharide standards in distilled water and preparing a mixed standard solution, in which the concentration of each monosaccharide was 2 mmol/L.

### 2.4. In Vitro Digestion of PFP

Based on the INFOGEST protocol, the in vitro digestion characteristics of PFP were investigated, and a 1.25 × electrolyte stock solution was prepared [18]. In vitro, salivary simulated digestive fluid (SSF), gastric digestive fluid (SGF), and small intestinal digestive fluid (SIF) consisted of a four-fold volume of 1.25 × electrolyte stock solution mixed with a one-fold volume of distilled water [18,19,20]. The in vitro digestion scheme is shown in Figure 1B.

#### 2.4.1. In Vitro Saliva Digestion

Initially, SSF was prepared by adjusting the pH to 7.0 using 1 mol/L NaOH, followed by the addition of a suitable quantity of α-amylase to reach a final enzyme activity of 75 U/mL. Taking 10 mL of SSF mixed with 10 mL of PFP polysaccharide solution (5 mg/mL), this was set as experimental group A; taking 10 mL of PFP mixed with 10 mL of ultrapure water, this was set as experimental group B; and taking 10 mL of SSF mixed with 10 mL of ultrapure water, this was set as experimental group C. All experimental groups were incubated in a water bath set to 37 °C for the digestion process. At both 0 min and 2 min, 5 mL of the digest were extracted and quickly transferred to a boiling water bath at 100 °C for 10 min to inhibit enzymatic activity, followed by centrifugation at 8000 rpm for an additional 10 min. Each set of experiments was repeated three times and the collected samples were stored in a −20 °C refrigerator for backup.

#### 2.4.2. In Vitro Gastric Digestion

We prepared SGF using previously formulated 1.25 × electrolyte stock solutions. The pH was adjusted to 3.0 with 1 mol/L of HCl, and appropriate amounts of lipase and pepsin were taken to achieve final enzyme activities of 60 U/mL and 2000 U/mL. The polysaccharide that underwent saliva digestion had an adjusted pH of 3.0 and was mixed with SGF at 1:1, and 3 experimental groups were set up: Group A (10 mL saliva digest + 10 mL SGF); Group B (10 mL ultrapure water + 10 mL saliva digest); and Group C (10 mL ultrapure water + 10 mL SGF). All experimental groups were incubated in a water bath set to 37 °C for the digestion process. At 0 h, 1 h, and 2 h, 5 mL of digested solution were removed and immediately placed in a boiling water bath at 100 °C for 10 min and centrifuged.

#### 2.4.3. In Vitro Small Intestinal Digestion

Similarly, SIF was created by mixing fresh bile with 50 g of a 7% pancreatic enzyme solution (*w*/*v*) and trypsin to reach a final enzyme activity of 100 U/mL. The pH was adjusted to 7.0 using a 0.1 mol/L NaOH solution. The gastric digest from the previous phase was adjusted to pH 7.0 and thoroughly mixed with SIF in a 1:1 ratio to establish three experimental groups: Group A (10 mL gastric fluid digestive solution + 10 mL SIF); Group B (10 mL ultrapure water + 10 mL gastric fluid digestive solution); and Group C (10 mL ultrapure water + 10 mL SIF). Digestion was carried out in a 37 °C water bath. At 0 h, 1 h, and 2 h, 5 mL of the digest were withdrawn and immediately placed in a boiling water bath at 100 °C for 10 min and then centrifuged.

### 2.5. Determination of Physicochemical Properties of PFP During Digestion In Vitro

A total of 2 mL of the PFP digestive fluid at various stages of digestion were sampled and filtered through a 0.22 μm membrane. The Mw of each digestion product was determined, as previously described. The determination of reducing sugars was carried out using the 3,5-dinitrosalicylic acid method [21].

### 2.6. In Vitro Simulated Fecal Fermentation

In vitro fecal fermentation methods were measured according to the reported method with minor modifications [22,23,24]. The in vitro fecal fermentation scheme is shown in Figure 1B.

To begin with, a basic nutrient medium was prepared. A total of 1.0 g of yeast paste, 1.0 g of peptone, 0.25 g of bile salt, 0.25 g of L-cysteine, 0.05 g of NaCl, 0.02 g of K_2_HPO_4_, 0.005 g of CaCl_2_-6H_2_O, 0.005 g of MgSO_4_-7H_2_O, 1.0 g of NaHCO_3_, 0.01 g hemoglobin, 0.05 mg of resin cyanine, 1.0 mL of Tween 80, and 5 μL of vitamin K were added to 500 mL of ultrapure water. The pH was adjusted to 7.0 with 0.1 M HCl and sterilized at 121 °C for 15 min.

A specific volume of fresh feces was obtained from six healthy volunteers during the same timeframe, consisting of three males and three females aged between 20 and 30 years. None had consumed antibiotics or probiotics in the past three months. Equal portions of mixed feces were diluted with saline (NaCl at 9.0 g/L) to achieve a pH of 7.0, resulting in a fecal suspension at a concentration of 10% (*w*/*v*). The fecal suspension was filtered using sterile gauze and the filtrate was taken as human fecal microbial inoculum. Three experimental groups were set up and the mixed microbial suspensions were inoculated into the culture medium of each group separately. The Blank group consisted of 1 mL of bacterial suspension combined with 9 mL of medium. The inulin group (INL) included 1 mL of bacterial suspension mixed with 9 mL of medium containing 100 mg of inulin. The PFP group was composed of 1 mL of bacterial suspension and 9 mL of medium that contained 100 mg of PFP. All operations were carried out under nitrogen protection and the inoculated medium was placed in an anaerobic incubator. The fermentation broth of each group was removed at 0, 6, 12, 24, and 48 h of fermentation at 37 °C for testing and analysis, and each group of experiments was repeated three times. After stopping fermentation, centrifugation was performed at 5000 rpm for 10 min at 4 °C. The resulting precipitate was stored at −80 °C for microbiological analysis, while the supernatant was utilized to assess other parameters.

### 2.7. Determination of Physical and Chemical Properties of PFP During In Vitro Fermentation

#### 2.7.1. Changes in Basic Composition, Mw, and Monosaccharides

The total soluble sugar, reducing sugar, uronic acid, and Mw of each group of fermentation broths were determined according to the previous method. The Mw and free monosaccharide content were measured.

#### 2.7.2. Determination of pH and SCFAs

The pH of the fermentation products at different times in each group was measured using a pH meter. According to the previously reported method, SCFAs in the fermentation culture were determined by gas chromatography [22,25]. Chromatographic analysis was performed using a gas chromatograph (7820A Agilent, Santa Clara, CA, USA) fitted with an Agilent DB-FFAP column (30 m, 0.25 mm, 0.25 μm) (Agilent Technologies, Santa Clara, CA, USA). The GC analysis conditions were as follows: flame ionization detector, carrier gas N_2_ flow rate of 30.0 mL/min, carrier gas air flow rate of 270 mL/min, and carrier gas H_2_ flow rate of 40 mL/min. The detector temperature was 240 °C and the inlet temperature was 230 °C. The warming program was 90 °C (0.5 min) to 130 °C (5 °C/min), and finally held at 220 °C for 5 min at a rate of 15 °C/min. The injection volume of the sample was 1 µL, the split ratio was 1:10, and the time for each determination was 20 min. All results are expressed in mmol/L.

#### 2.7.3. Analysis of Gut Microbiota

Fermentation broth from the Blank, INL, and PFP groups fermented for 48 h was taken and centrifuged at 10,000 rpm for 10 min to collect the flora, and DNA was extracted using a DNA extraction kit (FastDNA® Spin Kit for Soil, MP Biomedicals, Santa Ana, CA, USA). The microbiological composition of the extracted samples was analyzed by Shanghai Majorbio Bio-Pharm Technology Co., Ltd. (Shanghai, China), and the 16s bacterial rRNA genes in the V3–V4 region were amplified by PCR using universal primers and sequenced by Illumina Miseq PE300 using high-throughput sequencing. Data analysis was conducted on the I-Sanger cloud platform https://cloud.majorbio.com (accessed on 6 January 2025). The metrics analyzed include the operational taxonomic units (OTU), statistical analysis and annotation, species abundance statistics and community composition analysis, Alpha and Beta Diversity Index analysis, and Principal Component Analysis (PCoA) among the samples.

#### 2.7.4. Metabolomics Analysis

Shanghai Majorbio Bio-Pharm Technology Co., Ltd. (Shanghai, China) conducted the determination of metabolites in the 48-hour fermentation broth. Detailed information regarding the specific methodology can be found in the referenced report [26]. Liquid chromatography–mass spectrometry (LC-MS) analysis was performed using an ExionLC AD liquid chromatography system (AB SCIEX, USA) coupled with a UPLC HSS T3 column (100 mm × 2.1 mm i.d., 1.8 µm) and a triple quadrupole mass spectrometer (Triple TOF 6600, AB SCIEX, Redwood City, CA, USA). The instrument platform for this LC-MS analysis was provided by Shanghai Majorbio Bio-Pharm Technology Co., Ltd. (Shanghai, China). Briefly, 200 µL of sample were taken and 800 µL of extraction solution (methanol/acetonitrile = 1:1 (*v*/*v*)) containing four internal standards (0.02 mg/mL L-2-chlorophenylalanine) were added. After vortexing, ultrasonic extraction was carried out at 5 °C with a frequency of 40 KHz for 30 min. The sample was then placed at −20 °C for 30 min to deproteinize, and centrifuged at 12,000 rpm at 4 °C for 15 min. The sample was dried under nitrogen. After re-dissolving with 120 μL of 50% acetonitrile, the above operation was repeated. The supernatant was transferred to a sample vial and analyzed by LC-MS. The chromatographic conditions were as follows: the chromatographic column was ACQUITY UPLC HSS T3 (100 mm × 2.1 mm, 1.8 µm; Waters, Milford, MA, USA); mobile phase A was 95% water + 5% acetonitrile (containing 0.1% formic acid), mobile phase B was 47.5% acetonitrile + 47.5% isopropanol + 5% water (containing 0.1% formic acid); the injection volume was 3 μL; and the column temperature was 40 °C. The mass spectrometry conditions were as follows: Samples were ionized by electrospray ionization. Mass spectra were collected in both positive and negative ion scanning modes, with a mass scanning range of 50–1200 m/z. Raw data were processed using the Progenesis QI v3.0 software (Waters Corporation, Milford, MA, USA) to generate a data matrix. Subsequently, metabolites were identified by referring to the HMDB database (Human Metabolome Database http://www.hmdb.ca/ (accessed on 15 January 2025)) and the Metlin database (METLIN|Scripps Research https://metlin.scripps.edu/ (accessed on 15 January 2025)). Based on the KEGG database (http://www.genome.jp/kegg/ (accessed on 15 January 2025)), differential metabolites between the two groups were selected for metabolic pathway analysis.

### 2.8. Statistical Analysis

The experimental data were expressed as mean ± standard deviation (SD) and all the experiments were repeated three times. Statistical analyses were performed using IBM SPSS Statistics 27. Differences between means were assessed using one-way analysis of variance (ANOVA) and Tukey’s test to determine the statistical significance of the data. *p* < 0.05 was defined as statistically significant.

## 3. Results and Discussion

### 3.1. Physical and Chemical Properties of PFP

**Figure 2:**
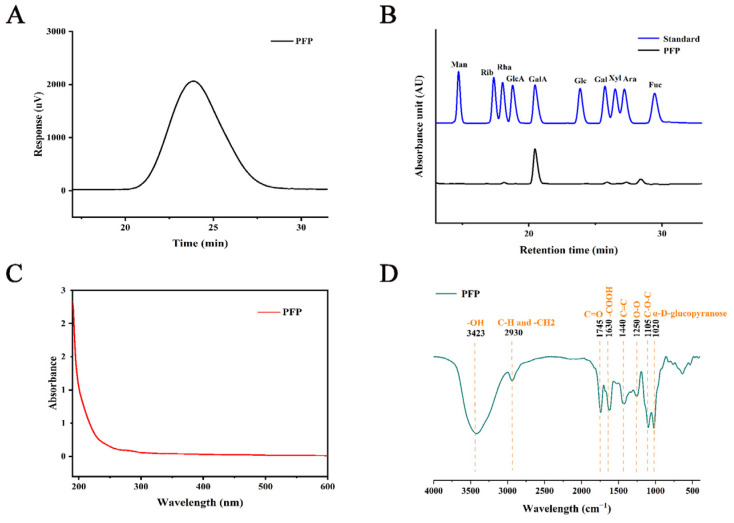
(**A**) HPGPC chromatogram of PFP, (**B**) HPLC analysis of the monosaccharide composition of PFP and the standard product, (**C**) UV spectra of PFP, and (**D**) FT-IR spectra of PFP.

The elution curves for PFP isolation and purification are shown in Figure S1. The HPGPC graph of the purified pectin showed a symmetric normal single peak, indicating that PFP is a pectin of uniform Mw and high purity (Figure 2A). Based on the standard Mw curve of the dextran standard, the average Mw of PFP was calculated to be 851.25 kDa, which is a typical biological macromolecule. Secondly, the high-performance liquid chromatography (HPLC) results showed that the monosaccharide composition of PFP mainly included galacturonic acid (GalA, 82.53%), arabinose (Ara, 7.00%), galactose (Gal, 4.89%), rhamnose (Rha, 3.59%), and glucose (Glc, 1.99%) (Figure 2B). These results differ from previous findings and may be related to the separation and purification process [6]. 

The research findings presented in Table 1 demonstrated that the total soluble sugar content of PFP was 95.69 ± 1.9% and the uronic acid content was 70.04 ± 1.75%. These results indicated that PFP was a pure acidic pectin polysaccharide with a high uronic acid content, aligning with the significant GalA content in its monosaccharide composition. The results of the UV spectral analysis, depicted in Figure 2C, did not reveal a peak at 280 nm, suggesting the absence of proteins in PFP. Furthermore, the FT-IR spectra (Figure 2D) showed the functional groups present in PFP, with the peak at 3423 cm^−1^ corresponding to the stretching vibration of -OH and the weak absorption peak at 2930 cm^−1^ indicating the asymmetric stretching vibrations of C-H and -CH_2_ [27]. The peak at 1745 cm^−1^ was the main characteristic absorption of the ester group, which was a C=O stretch. The strong absorption peak at 1630 cm^−1^ suggested that GalA was present in PFP, and the characteristic peak here belonged to the carboxyl-related products of polysaccharides containing -COOH. The peak at 1440 cm^−1^ belonged to the vibration of the C=C backbone, and the weak absorption peak at 1250 cm^−1^ came from the bending of the oxygen bridge (O-O), indicating the presence of uronic acid in PFP [28]. Furthermore, the vibrational stretch at 1105 cm^−1^ was associated with the C-O-C group, which suggested the presence of compounds related to the pyran ring in the polysaccharide, and the characteristic peak at 1020 cm^−1^ indicated the presence of the α-D-glucopyranose structure in PFP [29].

### 3.2. Simulated Digestive Characteristics of PFP In Vitro

#### 3.2.1. Variations in Reducing Sugars

Like other dietary fibers, pectin travels to the distal colon, fermented by the gut microbiota, resulting in positive impacts on the human body [30]. Nonetheless, the specific luminal conditions and pH levels in the upper gastrointestinal tract may influence these heteropolysaccharides prior to their arrival in the colon, potentially resulting in chemical and physicochemical alterations that impact both the rate and degree of colonic fermentation [31]. As a result, changes in reducing sugars serve as a key indicator for assessing the digestive characteristics of PFP. The changes in reducing sugar content of PFP during simulated digestion in vitro are shown in Table 2. There were no significant changes in the simulated oral digestion stage, a slight increase in reducing sugar content in the gastric stage, and no significant changes in reducing sugar in the small intestine stage, similar to the results of Ye et al. [24]. Another study has shown that pectin is a non-digestible carbohydrate and its degradation in the human gastrointestinal tract is only mediated by a small community of microorganisms in the human gut [32]. This demonstrates that PFP is relatively stable in the GI tract and is not easily degraded.

#### 3.2.2. Mw Analysis

**Figure 3:**
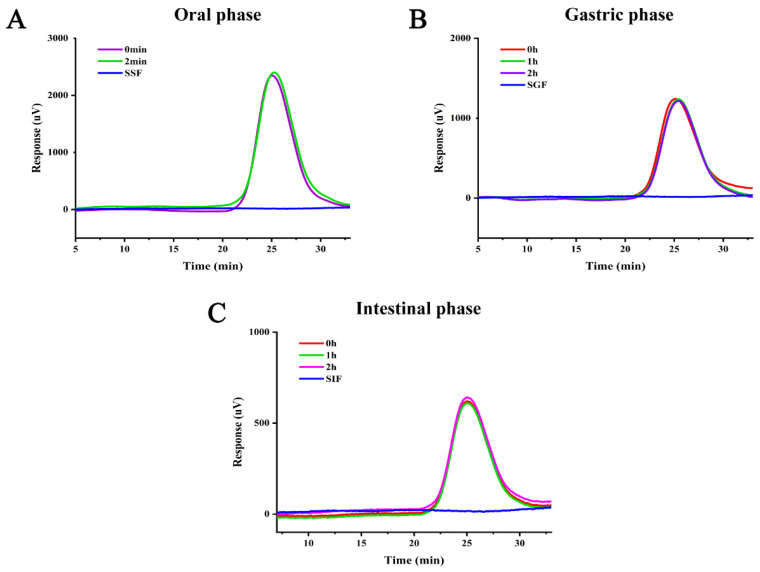
Molecular weight changes of PFP during oral (**A**), gastric (**B**), and intestinal (**C**) digestion.

The digestion of polysaccharides in the gastrointestinal tract has been shown to usually lead to a partial decrease in Mw, and the biological activity of polysaccharides is closely linked to their Mw and functional groups [33]. Therefore, the change in Mw is also an important indicator for evaluating the digestive properties of PFP. The changes in Mw of PFP during gastrointestinal digestion are shown in Figure 3A–C. As can be seen from Figure 3A, the two PFP curves after simulated saliva digestion at 0 and 2 min almost overlapped. Thus, PFP is invariant during the oral phase. Figure 3B shows that the results after digestion with simulated gastric fluid for 0–2 h were like those of simulated saliva. Figure 3C shows that the retention time of PFP did not change during digestion in the small intestine, suggesting that PFP remained stable during digestion in the salivary gastrointestinal tract. Similar studies on pectins such as peach gum polysaccharides and okra polysaccharides have previously been reported [23,34]. In summary, PFP as a pectin is resistant to gastrointestinal digestion and can arrive in the colon intact to participate in fermentation.

### 3.3. Characteristics of In Vitro Fermentation of PFP

#### 3.3.1. Change of Mw

**Figure 4:**
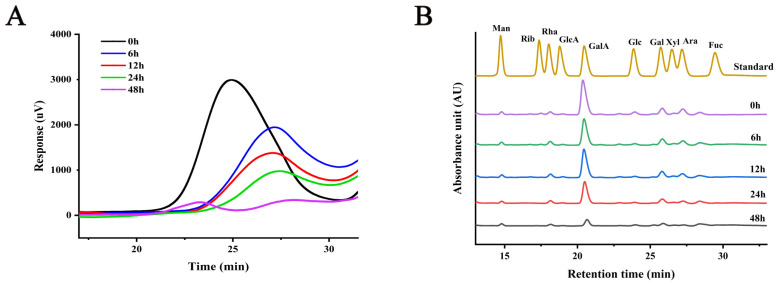
(**A**) HPGPC chromatogram of PFP in fermentation broth at different times in vitro; (**B**) changes of monosaccharide composition during in vitro fermentation of PFP at different times.

Figure 4A shows the HPGPC chromatogram of PFP in vitro with different fermentation times. As shown in Figure 4A, the retention time of PFP was delayed, and there was a significant reduction in peak area as the fermentation time increased. This suggests that the Mw of PFP decreased, and its abundance diminished, making it more susceptible to degradation by the gut microbiota. Especially at 6 h of fermentation, the characteristic peak of PFP significantly shifted to the right to 27 min, indicating that during this period, the intestinal microbiota could rapidly degrade PFP, breaking it down into small molecular fragments and thus destroying its glycosidic bonds. Specifically, PFP is rich in GalA with a large extended Mw conformation, which is a better carbon substrate and can be rapidly used by microbes during the initial phases of fermentation, and similar results have been reported [24,35,36]. Furthermore, the maximum area of PFP gradually decreased during the fermentation phase from 6 to 48 h, until it was almost completely degraded at 48 h, suggesting that PFP was used by the gut microbiota during the fermentation process.

#### 3.3.2. Carbohydrate Consumption During Fecal Fermentation

Figure 4B shows the monosaccharide composition of fermented PFP in vitro for different times. Changes in the monosaccharide content of the fermentation broth from 0 to 48 h were mainly GalA, Gal, and Ara. The peak areas of GalA, Gal, and Ara decreased significantly over time, with GalA consumed significantly more than the other sugars, indicating that intestinal microorganisms preferentially degrade and utilize GalA, followed by Ara, Gal, and Rha. Numerous microbial strains isolated from human feces have been discovered to possess various pectinolytic enzymes such as polygalacturonases, pectin methyl esterases, and both extracellular and cell-associated pectate lyases. These enzymes play a crucial role in the breakdown and utilization of pectins present in plant food products [37].

In addition, uronic acid, total soluble sugar, and reduced sugar contents in fermentation broths at different fermentation times are also believed to be useful in assessing the consumption of carbohydrates by the gut microbiota. As shown in Table 3, as the fermentation time extends, the residual amount of total soluble sugar and uronic acid of PFP gradually decreased, and the total soluble sugar content of the reducing sugar first increased and then decreased, which is consistent with the results of Wei et al. [34]. The remaining residual uronic acid, total soluble sugar, and reducing sugar content in the PFP fermentation broth remained high and slowly decreased during 12 h. During the fermentation process from 12 to 48 h, the rate of carbohydrate decline became faster due to the decrease in Mw. At 48 h, the total soluble sugar residues of the PFP group and the INL group were 26.47% and 22.79% of the initial values, respectively, which were closer to each other, and from this point of view, this indicated that PFP was as easily utilized by microorganisms as inulin.

#### 3.3.3. Change of pH and SCFAs

**Figure 5:**
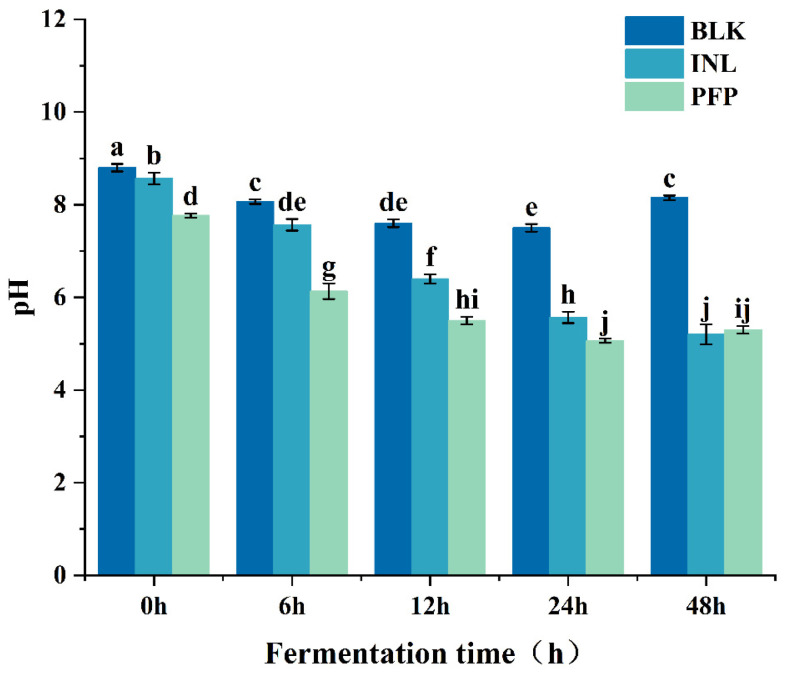
Changes in pH of PFP and inulin sugar at different fermentation times. Different letters indicate significant differences in pH (*p* < 0.05).

The variation in pH serves as a crucial parameter for tracking the polysaccharide fermentation process. As illustrated in Figure 5, at the beginning of fermentation (0 h), the initial pH of the fermentation broth in the Blank, INL, and PFP sample groups exceeded 7, which aligns with the results reported by Fang et al. [19]. The Blank and INL groups had the highest pH of the initial fermentation broth. In contrast, the pH of the initial fermentation broth of the PFP group decreased slightly as a result of the addition of the samples, which may be related to the higher uronic acid content in PFP. During the first 24 h of fermentation, the pH in the PFP group decreased from 7.4 to 5.0, showing a significant drop (*p* < 0.05) within this time frame, with a smaller change observed at 48 h. On the contrary, the pH in the INL group gradually decreased from 8.5 to 5.2, with ΔpH = 3.3. The Blank group experienced a slight decrease in pH from 0 to 24 h, stabilizing around 8.0 thereafter. Throughout the fermentation process, the pH of the PFP and INL groups remained consistently lower than that of the Blank group. This revealed that the presence of polysaccharides in colonic fermentation has a regulatory effect on intestinal pH.

The SCFA content of each group at the end of fermentation is shown in Table 4. The Blank group had the lowest levels of major gut flora metabolites such as acetic acid, propionic acid, and *n*-butyric acid, which were 8.12, 4.16, and 1.38 mmol/L, respectively. The levels of these three SCFAs were significantly elevated in both the INL and PFP groups. The contents of acetic acid, propionic acid, and *n*-butyric acid in the INL group were 14.29, 6.50, and 8.93 mmol/L, respectively. The PFP group had the highest levels of acetic acid, followed by propionic acid, and a smaller increase in *n*-butyric acid than the INL group, with 29.18, 6.92, and 3.24 mmol/L, respectively. This finding is similar to those that reported the highest concentrations of acetic acid, followed by propionic acid and *n*-butyric acid [38]. Furthermore, some studies have proposed an effect of sugar composition on SCFA production by fermentation, such as the fermentation of Xyl and GalA, that significantly increases the content of acetic acid and *n*-butyric acid [39]. There is a positive correlation between propionic acid production and Ara, Rha, and Xyl content in pectin [12]. This suggests that the elevated propionic acid content in the PFP group in this study is related to the fermentation of Ara and Rha. On the contrary, the high production of acetic acid in the PFP group corresponded to the structural domains of pectin of the high HG type, suggesting that acetic acid is produced primarily by GalA fermentation by the gut microbiota, which is in agreement with the findings of Tian et al. [40]. Other plant polysaccharides also have similar effects. For instance, aloe polysaccharides significantly increased the levels of SCFAs such as acetic acid, propionic acid, and butyric acid in the fermentation broth, and could act as signal molecules on the “gut–liver” axis to regulate fatty acid oxidation and immune cells in the liver [41]. Polysaccharides from yam can promote the production of lactic acid, acetic acid, propionic acid, and butyric acid, and regulate the intestinal microenvironment [42]. SCFAs are considered a beneficial metabolite of the gut microbiota due to their anti-inflammatory properties, effects on glucose and energy homeostasis, and enhanced insulin sensitivity [43]. Acetic acid, propionic acid, and *n*-butyric acid are the main SCFAs released by the gut microbiota that ferment dietary fiber and are considered beneficial for human health. Acetic acid regulates intestinal pH and protects against pathogens. Propionic acid regulates cholesterol metabolism and fights inflammation. The *n*-butyric acid has significant immune and anti-inflammatory properties [44]. Moreover, SCFAs also serve as an energy source for intestinal epithelial cells [45]. The findings regarding the total SCFA content revealed that the PFP groups had higher levels of total SCFAs compared to the Blank and INL groups. This suggests that incorporating PFP can significantly enhance SCFA levels, which is advantageous for human health.

#### 3.3.4. Effects of PFP Fermentation on Intestinal Microorganisms

##### Microbial Diversity Analysis

**Figure 6:**
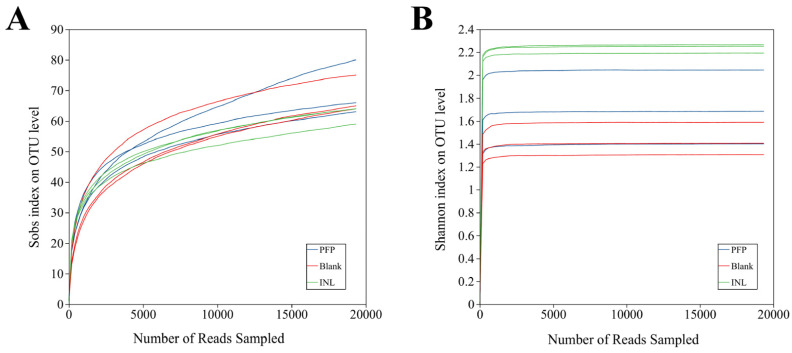
Microbial α diversity dilution curve: (**A**) Sobs, and (**B**) Shannon.

Alpha diversity refers to the diversity within a specific region or ecosystem and is a measure of the species richness and community diversity of microorganisms in a sample. Shannon indices are commonly used to assess this diversity, with higher Shannon values indicating greater species diversity. The Sobs index was used to predict the number of microorganisms and measure the richness of the community, with larger values indicating more species. As shown in Figure 6A,B, their dilution curves reflect the sample size and sequencing depth. Both dilution curves plateau, indicating that the sequencing data have reached saturation and are able to cover most species in the gut microbiota. In addition, it is evident that as the levels of the Sobs and Shannon indices increase, bacterial α diversity also rises. The Shannon values for both the PFP and INL groups were significantly higher than those observed in the Blank group, indicating that PFP and inulin substantially altered the species diversity within the microbiota. Generally, an increase in gut microbial diversity correlates with a decreased risk of disease; thus, it can be concluded that the incorporation of PFP mitigates the likelihood of disease occurrence [46].

**Figure 7:**
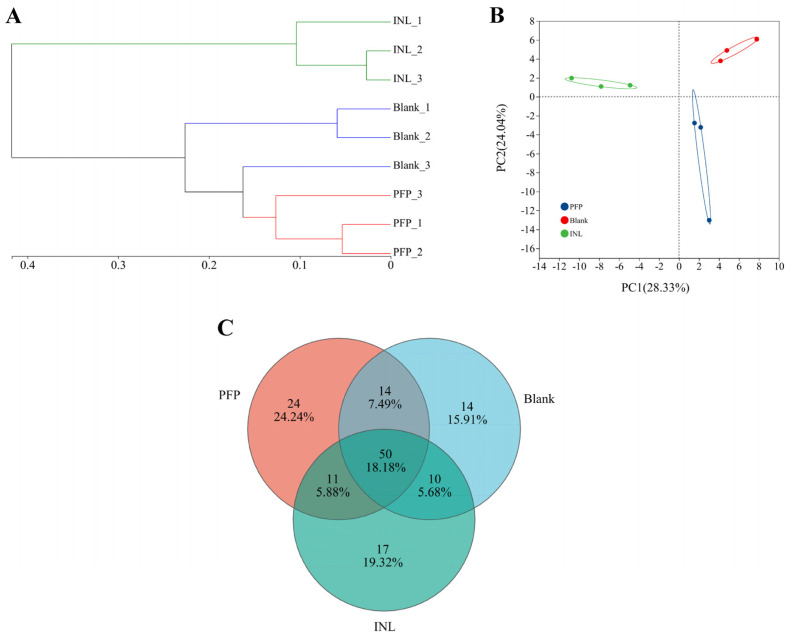
Microbial β diversity analysis: (**A**) sample level clustering, (**B**) PCA analysis, and (**C**) Venn diagram.

In addition, beta diversity, also referred to as interhabitat diversity, pertains to the variation in species composition among different habitat communities or the rate of species turnover along environmental gradients. In this study, hierarchical cluster analysis, principal coordinate analysis (PCA), and Venn diagrams were employed to elucidate the overall differences in the gut microbiota across the three groups. Hierarchical cluster analysis illustrates sample similarity through a dendrogram format and assesses clustering effectiveness based on the branch lengths of the resulting tree. As shown in Figure 7A, the INL group was a cluster by itself, which was significantly different from the other groups. The PFP group and Blank group are a cluster, indicating that some gut microbiota in the PFP group may be similar to those in the Blank group. PCA was employed to assess overall differences in the gut microbiota among the three groups. The results showed that the distance between the PFP group, INL group, and Blank group was far, and there was statistical separation among the groups (Figure 7B). The cumulative variance contribution rate of the PC1 and PC2 principal component factors is 52.37%, indicating that most of the information in each group can be explained. Furthermore, a Venn diagram was employed to assess the number of OTUs across each group. As illustrated in Figure 7C, the PFP group, INL group, and Blank group exhibit unique OTU counts of 24, 17, and 14, respectively, and the number of OTUs in the PFP group is significantly higher than those in the other two groups, indicating that the gut microbiota structure has undergone significant changes under the treatment of PFP.

##### Effect of PFP on the Structure of the Gut Microbiota

**Figure 8:**
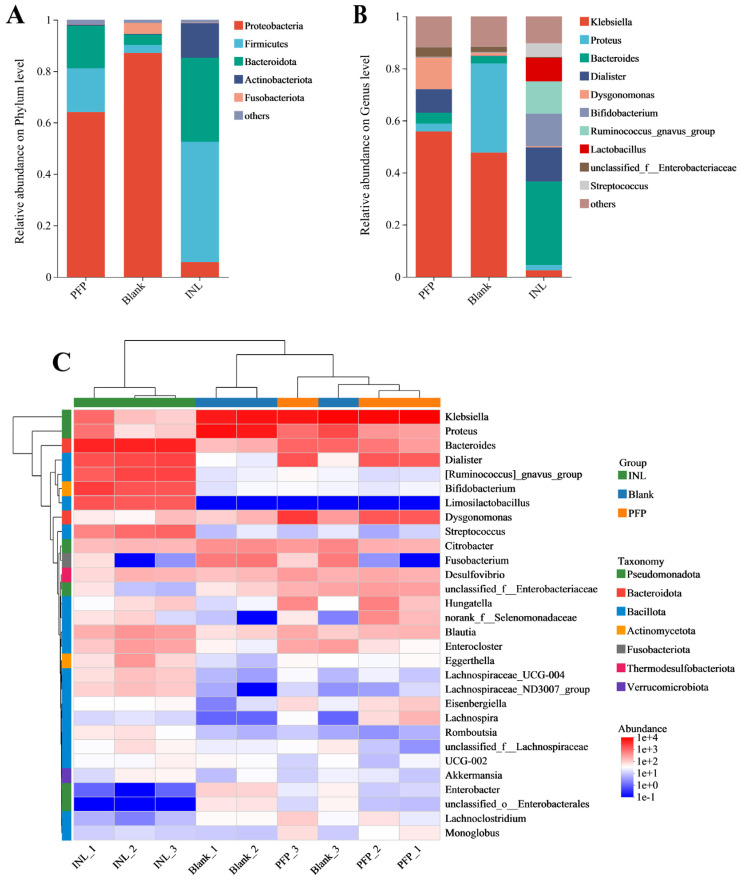
Microbial composition at the (**A**) phylum and (**B**) genus level, and (**C**) changes in the most abundant microbiota at the genus level.

Initially, we evaluated the microbial community structure of each group at the phylum level, with the results presented in Figure 8A. After 48 h of fermentation, significant changes were observed in the gut microbiota of both the PFP and INL groups. Compared to the Blank group, there was a notable increase in the abundances of *Bacteroidota*, *Firmicutes*, and *Actinomycetota* within the INL group (*p* < 0.05). Conversely, the abundances of *Proteobacteria* and *Fusobacteriota* were significantly reduced (*p* < 0.05). Similarly, in the PFP group, there was a marked increase in *Bacteroidota* and *Firmicutes* abundance (*p* < 0.05), while *Proteobacteria* and *Fusobacteriota* showed a significant decrease (*p* < 0.05). These findings suggest that PFP and inulin exhibit certain similarities regarding their regulatory effects on the gut microbiota at the phylum level. Studies have demonstrated that an abnormal increase in *Proteobacteria* is closely linked to a significant accumulation of endotoxins, which not only disrupts intestinal barrier function but may also exacerbate the progression of diabetes [47]. Furthermore, an elevation in the phylum *Fusobacteriota* is frequently regarded as a microbial marker indicative of malnutrition [48]. *Bacteroides* play an important role in the gut microbiota, encoding a series of glycosidases and polysaccharide lyases, which constitute a system similar to the sugar utilization system (SUS). The presence of these enzymes allows *Bacteroides* to efficiently break down and utilize complex carbohydrates, including biomacromolecules such as pectin [49]. In *Bacteroides thetaiotaomicron* and *Bacteroides ovatus*, genes for carbohydrate active enzymes (CAZymes) make up about 6% of the genome, while most organisms account for only 1–3% [50]. This indicates that *Bacteroides* have a strong advantage in the degradation and utilization of polysaccharides.

Secondly, to elucidate the regulatory effect of PFP on the microbial community, we evaluated the intestinal microbiota of the PFP, INL, and Blank groups following 48 h of in vitro fermentation at the genus level. The results are presented in Figure 8B. Compared to *Proteus* and *Klebsiella*, which constituted over 50% of the microbial population in the Blank group, the relative abundance of *Proteus* in both the PFP group and INL group decreased to below 5%. The relative abundance of *Klebsiella* in the INL group also decreased to less than 5%. At the same time, *Bacteroides* and *Dialister* in the two groups increased by different degrees. This finding indicates that PFP and inulin significantly altered the distribution of microorganisms. Figure 8C shows the results of bacterial cluster analysis in the different experimental groups, revealing the differences in bacterial composition among the different groups. The depth of the color represents the relative abundance change of the genus, and the darker the color, the higher the relative abundance. Compared with the Blank group, the abundance of *Bacteroides*, *Dialister*, *Dysgonomonas*, and other beneficial bacteria in the PFP group increased, while the abundance of conditioned bacteria such as Proteus decreased significantly. The INL group significantly increased the abundance of *Bacteroides*, *[Ruminococcus]_gnavus_group*, *Dialister*, *Limosilactobacillus*, and *Bifidobacterium*. It inhibited the growth of harmful bacteria such as *Proteus* and *Klebsiella*. Among them, *Bifidobacterium*, as an important gut beneficial bacterium, has a positive impact on human health in many aspects. It provides significant benefits to host health by improving the immune response and anti-tumor effects, and reducing susceptibility to allergies [51].

*Dysgonomonas* is a genus in the phylum *Bacteroides* that is rich in carbohydrate esterases (CEs), which play an important role in the intestinal cleavage of dietary fibers such as polysaccharides [52]. *Dialister* is a genus of *Veillonellaceae* that metabolizes complex carbohydrates and produces SCFAs such as acetic acid, propionic acid, and butyric acid [53]. Studies have shown that the addition of cereals can increase the relative abundance of the fiber-degrading bacteria *Veillonellaceae* in the digestive tract of infants, and the presence of these bacteria is positively correlated with acetic acid content, which is of great significance for intestinal health [54]. In addition, the abundance of *Dialister* in the gut correlates with levels of serotonin, which has inhibitory effects on neuropsychiatric disorders such as depression, autism, and mood control [55]. Meanwhile, low Mw longan pulp polysaccharides can increase the abundance of *Dialister* [56]. These findings suggest that the addition of PFP may exert its health benefits by promoting the growth of beneficial bacteria such as *Dialister* and *Bacteroides* and increasing the production of SCFAs.

**Figure 9:**
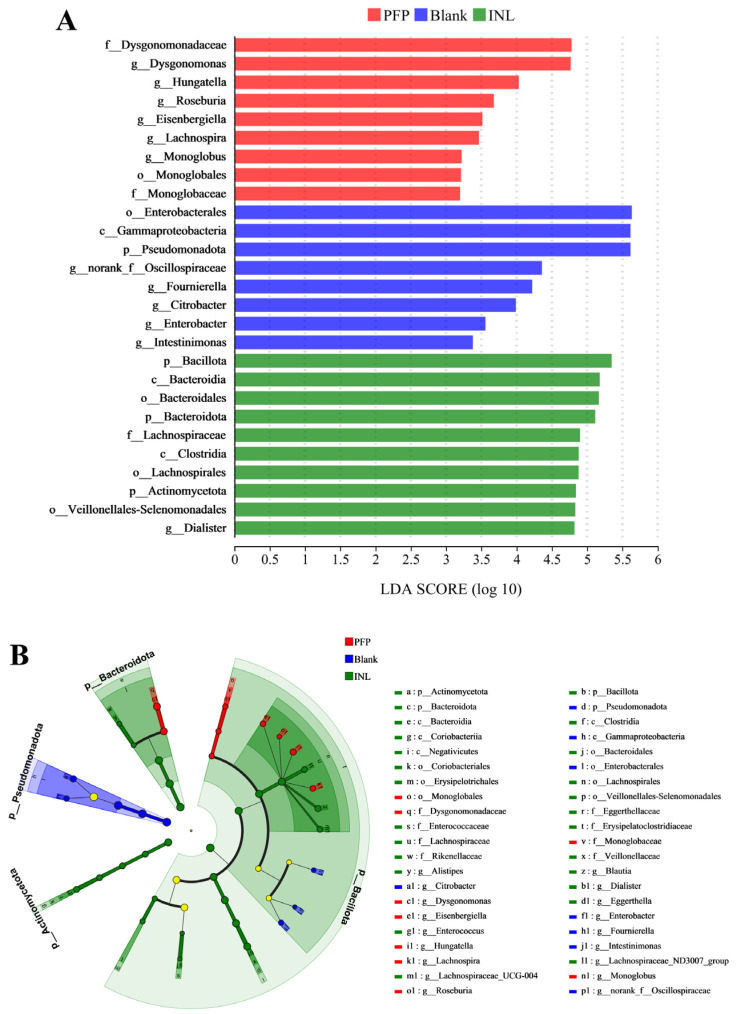
LEfSe showed significant differences in microbiota between the different groups. (**A**) LDA score; (**B**) LEfSe multilevel species hierarchy tree. Note: Yellow dots indicate species without significant differences.

Impact factor analysis and linear discriminant analysis (LDA) of multilevel species differences were performed using the linear discriminant analysis effect size (LEfSe) (Figure 9A). Bars of different colors were used to indicate species differences between groups with LDA scores (Log10) > 2 and significant abundance. The length of the bar is the LDA score, indicating the effect of significantly different species between groups. There were 8, 9, and 10 cases of OTUs in the Blank, PFP, and INL groups, respectively, and the differences were statistically significant. Among them, *g_Dysgonomonas*, *g_Hungatella*, *g_Roseburia*, *g_Lachnospira*, and *g_Monoglobus* are the dominant flora of the PFP group and can be used as potential biomarkers of the PFP group. A study isolated a *Firmicutes* strain, *H. hathewayi* N2-326, which can catabolize the complex polysaccharide glycosaminoglycan and is a rich source of specific glycosaminoglycan catabolic enzymes [57]. *Roseburia* is a genus of gut bacteria capable of breaking down complex carbohydrates, mainly producing SCFAs such as butyrate, which play an important role in maintaining intestinal health and alleviating intestinal inflammation [58]. As the main energy source of colon epithelial cells, butyrate can reduce the pH value of the colon, stimulate the proliferation of normal epithelial cells, inhibit the proliferation of colorectal cancer cells, and induce their apoptosis [59]. In addition, butyrate also improves intestinal barrier function and reduces the oxidative stress response by promoting the formation of mucin, antimicrobial peptides, and tight junction proteins, thus inhibiting the occurrence and development of colitis and colon cancer [59]. Polysaccharides from *Naematelia aurantialba*, a mushroom species, have been proven to enhance the abundance of *Roseburia* [60]. *Lachnospira*, a member of the phylum *Firmicutes*, has been noted for its ability to ferment dietary fibers such as pectin [61]. It plays an important role in carbohydrate metabolism, providing nutrients and energy to the host primarily through the production of butyrate, and is therefore considered a potential probiotic [61]. In addition, *Monoglobus*, a novel pectolytic bacterium recently isolated from human feces, is a beneficial microbe with the ability to ferment galacturonic acid, xylose, and arabinose [62]. The results showed that the addition of PFP could significantly increase the abundance of these beneficial bacteria, and help to maintain intestinal homeostasis and promote intestinal health.

#### 3.3.5. Microbial Metabolomics

**Figure 10:**
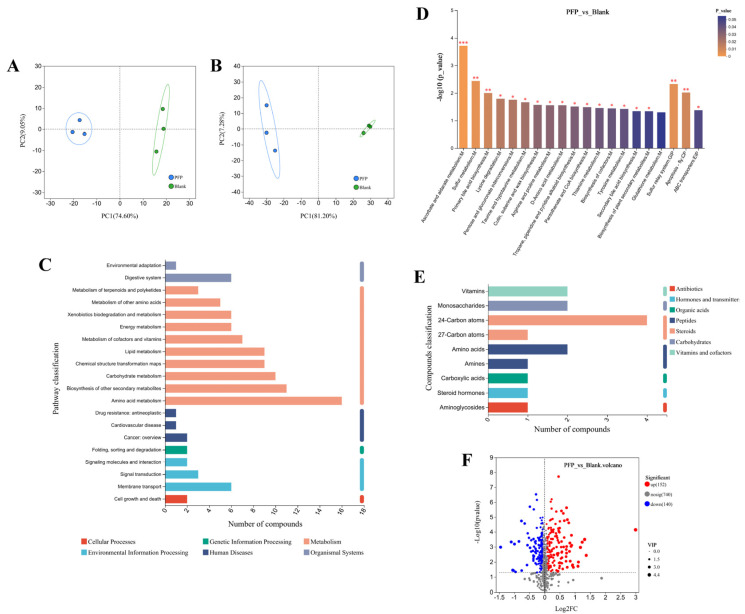
Metabolite PCA analysis, showing the (**A**) positive ion model and (**B**) negative ion model; (**C**) KEGG functional pathway analysis; (**D**) differential metabolites of PFP vs. Blank; (**E**) differential metabolite categories based on KEGG pathways; (**F**) volcano plot of differential metabolites. *, *p* < 0.05; **, *p* < 0.01; ***, *p* < 0.001.

The metabolic profiles of the fecal microbiota were systematically investigated by non-targeted metabolomics techniques using LC-MS. After data preprocessing and multivariate statistical analysis, 609 metabolites were successfully identified under positive ion mode (POS), 423 metabolites were detected under negative ion mode (NEG), and a total of 1032 metabolites were qualitatively and quantitatively obtained. The results of PCA analysis in Figure 10A,B showed that the PFP intervention group and the Blank control group showed significant separation under both ionization modes, in which the cumulative variance contribution rate of PC1 and PC2 reached 83.65% (POS) and 88.48% (NEG), respectively, fully reflecting the significant differences in metabolites between samples. This result suggests that PFP intervention may affect host–microbial co-metabolic processes by regulating the metabolic network of gut microbes, thus producing specific metabolic characteristics.

Through the pathway enrichment analysis of differential metabolites based on the Kyoto Encyclopedia of Genes and Genomes (KEGG) database, the significant differences in metabolic characteristics among different samples can be deeply understood. As shown in Figure 10C, the regulatory effect of PFP on the intestinal microbiota involves multiple metabolic pathways, covering major categories such as organismal systems, metabolism, human diseases, genetic information processing, environmental information processing, and cellular processes. Under the main category of metabolism, there are ten secondary classifications, including terpenoid and polyketide metabolism, amino acid metabolism, energy metabolism, lipid metabolism, carbohydrate metabolism, and cofactor and vitamin metabolism. Among them, lipid metabolism regulates the biosynthesis of primary bile acids, secondary bile acids, and steroid hormones; and amino acid metabolism regulates the synthesis of essential amino acids such as threonine, cysteine, lysine, valine, leucine, and isoleucine. In addition, PFP also exists in the regulation of metabolic pathways including butyric acid, vitamin B6, thiamine, and pantothenic acid. This indicates that PFP has a significant regulatory effect on the metabolic activities of the intestinal microbiota and can alter the metabolic products generated by bacteria. Figure 10D shows the statistical differences in metabolic pathways, and the results indicate that pathways such as ascorbate and aldarate metabolism, sulfur metabolism, and primary bile acid biosynthesis are significantly regulated by PFP (*p* < 0.01). Numerous studies have shown that plant polysaccharides can exert their biological activities by regulating specific metabolic pathways [49,63,64]. Taking Astragalus polysaccharides as an example, they can normalize the fasting blood glucose and insulin levels of high-fat diet mice by regulating the glutathione metabolic pathway and the purine metabolic pathway, thereby exerting significant protective effects against non-alcoholic fatty liver disease and obesity [65]. In terms of the repair of intestinal–liver–kidney axis injury, the polysaccharides of *Polygonatum sibiricum* have demonstrated unique metabolic regulatory functions, significantly influencing the pathways related to energy metabolism in the liver and kidneys, such as aspartic acid and tyrosine, and alleviating the intestinal flora disorder and liver and kidney damage caused by the co-exposure to citrinin and alcohol [66]. These research results collectively reveal the significant role of polysaccharides in maintaining the metabolic homeostasis of the body by interfering with the gut microbiota–host metabolic axis.

The differential metabolites of KEGG pathways were identified and classified. The classification statistics results are shown in Figure 10E. A total of seven types of differential metabolites were identified, including antibiotics, hormones and neurotransmitters, organic acids, peptides, steroids, carbohydrates, and vitamins and cofactors. The volcano plot can be used to analyze and compare the metabolites between the two groups by using the variable importance in projection (VIP), *p* value, and fold change (FC). Under the conditions of a *p* value < 0.05, VIP > 1, and FC > 1.2 or < 1/1.2, 292 differentially changed metabolites were screened out from 1032 major metabolites. As shown in Figure 10F, compared with the Blank group, 152 metabolites were significantly upregulated, and 140 metabolites were significantly downregulated in the PFP group. Based on the compound classification of the Human Metabolome Database (HMDB) (as shown in Figure S2), we have identified 12 categories of compounds, specifically including 56 organic acids and their derivatives, 50 lipids and lipid-like molecules, 38 organic heterocyclic compounds, 26 organic oxygen compounds, 20 benzene compounds, 12 phenylpropanoids and polyketides, four nucleosides, nucleotides, and their analogues, two with no information provided, one alkaloid and its derivatives, one lignan, neolignan, and related compounds, and one organic nitrogen compound. We selected several beneficial upregulated metabolites from those with VIP > 1, including vitamin B1 (thiamine), oxoadipic acid, S-hydroxymethylglutathione, leonuriside A, and isonicotinic acid. Thiamine is a water-soluble vitamin that is widely involved in energy metabolism and the maintenance of nervous system functions in the human body. It is absorbed in the upper part of the small intestine and converted into active forms such as thiamine pyrophosphate (TPP) and thiamine triphosphate (TTP) in the liver, serving as a cofactor for key enzymes in carbohydrate metabolism [67]. A deficiency of thiamine may lead to beriberi, neuritis, indigestion, and cardiovascular problems. Furthermore, the content of organic acids, such as oxoadipic acid in blueberries, is positively correlated with anti-cancer activity, which indicates that oxoadipic acid is beneficial to health [68]. Glutathione is a tripeptide composed of glutamic acid, cysteine, and glycine, and it has multiple physiological functions such as antioxidation, detoxification, and immune regulation. As an important endogenous antioxidant, glutathione is widely used in the food and pharmaceutical fields and can be used to delay aging and enhance immunity [69]. Research has found that leonuriside A not only possesses powerful antioxidant activity but also shows a significant inhibitory effect on the carcinogenic neurotoxin acrylamide, with its inhibitory effect even surpassing that of quercetin and catechin [70,71]. It has been reported that a series of isonicotinic acid derivatives were synthesized and their anti-tuberculosis activities were tested in vitro. Through a multi-target QSAR model, it was found that these derivatives have antibacterial activities against *Mycobacterium tuberculosis*, *Staphylococcus aureus*, *Escherichia coli*, etc. [72]. The fermentation metabolites of Atractylodes *chinensis* polysaccharides are related to energy metabolism and amino acid metabolism, significantly increasing the level of the insulin sensitizer myo-inositol [64]. Polysaccharides from *Rosa roxburghii* can stimulate the production of metabolites such as curcumin, 3-feruloylquinic acid, and citrulline after fermentation [49]. The above results indicate that PFP can regulate the metabolic activities of beneficial bacteria in the gut, generating probiotic metabolites with antioxidant and anti-inflammatory activities, thereby exerting beneficial effects on human health.

#### 3.3.6. Correlation Between Microbiota and Metabolites

**Figure 11:**
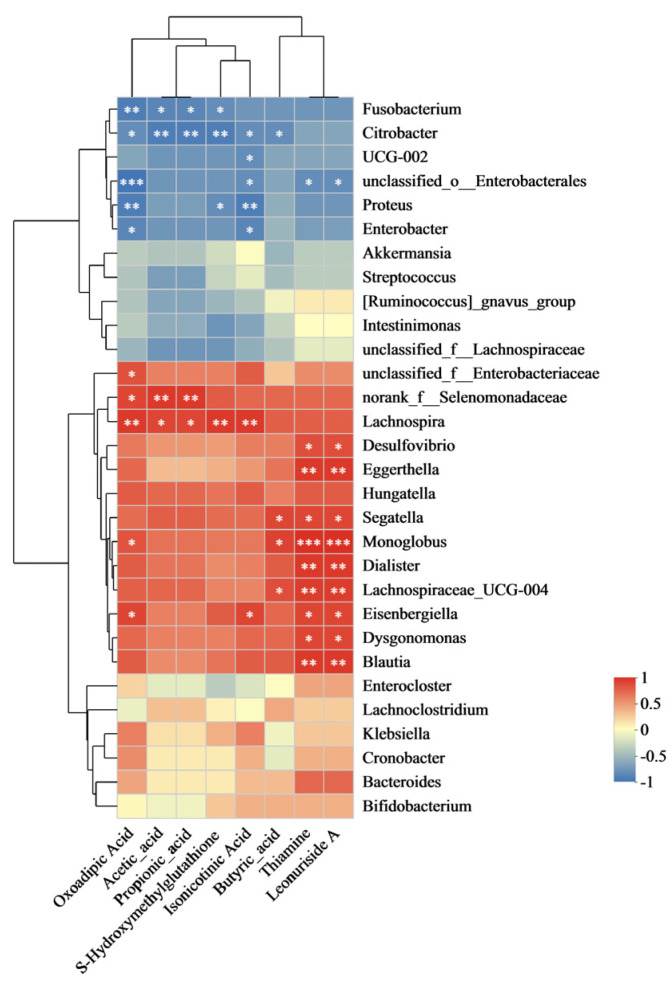
Correlation analysis of gut microbiota and metabolites. *, *p* < 0.05; **, *p* < 0.01; ***, *p* < 0.001.

Through Spearman correlation analysis, this study explored the correlations between the top 30 abundant microorganisms at the genus level and metabolites such as SCFAs and thiamine. As shown in Figure 11, the increased abundance of microorganisms such as *Monoglobus*, *Segatella*, and *Lachnospiraccae_UCG-004* was closely related to the elevated level of butyric acid. Additionally, the abundance changes of these microorganisms, along with those of *Dialister*, *Eisenbergiella*, *Dysgonomonas*, *Blautia*, and others, are significantly correlated with the elevated levels of differential metabolites such as thiamine and leonuriside A. *Lachnospira* and *norank_f_selenomonadaceae* mainly affect the production of acetic acid, propionic acid, and the metabolite oxoadipic acid. Meanwhile, *Lachnospira* is also related to the abundance of S-hydroxymethylglutathione and isonicotinic acid. The research results show that the intervention of PFP significantly enhanced the correlation between the microbiota and SCFAs as well as beneficial metabolic products, thereby promoting the healthy balance of the intestinal microbiota.

## 4. Conclusions

In this study, acidic pectin polysaccharide PFP was extracted and purified from *Pyracantha fortuneana* fruits by acid extraction and alcohol precipitation. The total soluble sugar content of PFP was 95.69%, and the uronic acid content was 70.04%. Its molecular weight was 851.25 kDa, which is that of a typical biological macromolecule. The results showed that PFP was not degraded during the simulated digestion process and could resist different digestive environments in the upper digestive tract, reaching the colon smoothly to participate in fermentation. The simulation fermentation results showed that, through the fermentation of PFP, the residual carbohydrate content and pH value of the fermentation broth were significantly reduced, while the contents of SCFAs such as acetic acid, propionic acid, and *n*-butyric acid were significantly increased. The intervention of PFP also promoted the proliferation of beneficial bacteria such as *Bacteroides*, *Dialister*, and *Dysgonomonas*, inhibited the growth of harmful bacteria like *Proteus*, and increased the concentrations of beneficial metabolites such as thiamine, leonuriside A, oxoadipic acid, S-hydroxymethylglutathione, and isonicotinic acid, which are beneficial to human health.

This study has found that PFP has great potential in generating SCFAs and regulating the composition of the intestinal microbiota and its metabolic products to promote intestinal functional health and can be used as a prebiotic. Furthermore, efforts will be devoted to in-depth exploration of the fine structure of PFP to reveal the structure–activity relationship between its structural characteristics and the intestinal microbiota. This research direction not only contributes to the analysis of the mechanism of action of PFP in the intestinal microenvironment but also provides a solid theoretical basis for the systematic study of intestinal functions and promotes further development in related fields.

## Figures and Tables

**Table 1:** Mw, physicochemical index, and monosaccharide composition of PFP.

PFP	Data
Mw (Da)	8.51 × 10^5^ ± 0.19
DM (%)	69.93 ± 0.17
Chemical composition (%)	
Total soluble sugars	95.69 ± 1.9
Uronic acid	70.04 ± 1.75
Monosaccharide composition (%)	
Rha	3.59 ± 0.06 ^d^
GalA	82.53 ± 0.24 ^a^
Glc	1.99 ± 0.10 ^e^
Gal	4.89 ± 0.08 ^c^
Ara	7.00 ± 0.01 ^b^

Note: Mw: molecular weight, DM: degree of methylation, Rha: rhamnose, GalA: galacturonic acid, Glc: glucose, Gal: galactose, Ara: arabinose. Different letters indicate significant differences (*p* < 0.05).

**Table 2:** Changes in reducing sugar content during simulated digestion of PFP in vitro.

Process	Time	Reducing Sugar (mg/mL)
Saliva digestion	0 min	0.0659 ± 0.0017 ^b^
	2 min	0.0679 ± 0.0010 ^b^
Gastric juice digestion	0 h	0.0859 ± 0.0004 ^a^
	1 h	0.0870 ± 0.0007 ^a^
	2 h	0.0870 ± 0.0001 ^a^
Small intestinal digestion	0 h	0.0463 ± 0.0004 ^c^
	1 h	0.0475 ± 0.0011 ^c^
	2 h	0.0492 ± 0.0008 ^c^

Different letters indicate significant differences (*p* < 0.05).

**Table 3:** Changes in the sugar content of PFP and inulin under different fermentation times.

Fermentation Time (h)	Remaining Total Soluble Sugar (% Initial)		Residual Uronic Acid (% Initial)	Reducing Sugar (mg/mL)
	PFP	INL		
0	100 ± 0 ^a^	100 ± 0 ^a^	100 ± 0 ^a^	0.24 ± 0.001 ^e^
6	80.91 ± 0.58 ^b^	95.67 ± 1.74 ^b^	77.59 ± 1.32 ^b^	0.29 ± 0.002 ^c^
12	80.16 ± 0.56 ^c^	88.04 ± 1.51 ^c^	69.23 ± 2.15 ^c^	0.33 ± 0.002 ^b^
24	59.46 ± 0.49 ^d^	53.57 ± 0.85 ^d^	62.91 ± 0.39 ^d^	0.36 ± 0.001 ^a^
48	26.47 ± 0.13 ^e^	22.79 ± 0.45 ^e^	48.07 ± 1.2 ^e^	0.27 ± 0 ^d^

Different letters indicate significant differences (*p* < 0.05). INL in the picture is inulin.

**Table 4:** Individual and total SCFA content of PFP after fermentation.

Sample	Acetic Acid (mmol/L)	Propionic Acid (mmol/L)	Isobutyric Acid (mmol/L)	Butyric Acid (mmol/L)	Isovaleric Acid (mmol/L)	Valeric Acid (mmol/L)	Total SCFAs (mmol/L)
Blank	8.12 ± 1.15 ^aD^	4.16 ± 0.48 ^bF^	0.98 ± 0.03 ^cH^	1.38 ± 0.22 ^cH^	0.81 ± 0.08 ^cH^	0.78 ± 0.05 ^cH^	16.23
INL	14.29 ± 0.41 ^aB^	6.50 ± 0.42 ^cE^	0.91 ± 0.00 ^dH^	8.93 ± 0.66 ^bC^	0.66 ± 0.02 ^dH^	0.78 ± 0.01 ^dH^	32.07
PFP	29.18 ± 0.64 ^aA^	6.92 ± 0.11 ^bE^	1.00 ± 0.01 ^dH^	3.24 ± 0.08 ^cG^	0.87 ± 0.02 ^dH^	1.23 ± 0.02 ^dH^	42.44

Analyzed in at least three replicates (*n* = 3). Lowercase letters indicate significant differences between samples in the same group (*p* < 0.05), and uppercase letters indicate significant differences between sample groups (*p* < 0.05).

## Data Availability

The original contributions presented in this study are included in the article/Supplementary Materials. Further inquiries can be directed to the corresponding author.

## Supplementary Materials

The following supporting information can be downloaded at https://www.mdpi.com/article/10.3390/foods14091529/s1: Figure S1: (A) PFP gradient elution curves of DEAE cellulose columns; (B) HPGPC profile during PFP purification. Figure S2: Compound classification of the Human Metabolome Database (HMDB).

## Abbreviations

The following abbreviations are mainly used in this manuscript:

Ara	Arabinose	Mw	Molecular weight
AGA	Apiogalacturonan	PFP	*Pyracantha fortuneana* polysaccharide
CAZymes	Carbohydrate active enzymes	PMP	1-pheny-3-methyl-5-pyrazolone
DEAE	DEAE cellulose DE-23	Rib	Ribose
FT-IR	Fourier transform infrared	Rha	Rhamnose
Fuc	Fucose	RG-I	Rhamnogalacturonan I
Glc	Glucose	RG-II	Rhamnogalacturonan II
GlcA	Glucuronic acid	ROS	Reactive oxygen species
Gal	Galactose	SSF	Simulated salivary fluid
GalA	Galacturonic acid	SGF	Simulated gastric fluid
SUS	Sugar utilization system	SIF	Simulated intestinal fluid
QSAR	Quantitative structure–activity relationship	LC-MS	Liquid chromatography–mass spectrometry
HPLC	High performance liquid chromatography	HPGPC	High-performance gel permeation chromatography
HG	Homogalacturonan	UV	Ultraviolet–Visible
IBD	Inflammatory bowel disease	Xyl	Xylose
INL	inulin	XGA	Xylogalacturonan
LDA	Linear discriminant analysis	SCFAs	Short-chain fatty acids
KEGG	Kyoto Encyclopedia of Genes and Genomes	HMDB	Human Metabolome Database
