# Supplementary material for: Simulated Gastrointestinal Digestion and In Vitro Fecal Fermentation of Purified Pyracantha fortuneana (Maxim.) Li Fruit Pectin
Source: Foods. 2025 Apr 27;14(9):1529. doi: 10.3390/foods14091529 (PMC12071275; doi:10.3390/foods14091529)
Supplement: Supplementary file 1 [file foods-14-01529-s001.zip › foods-3568727-supplementary.pdf]

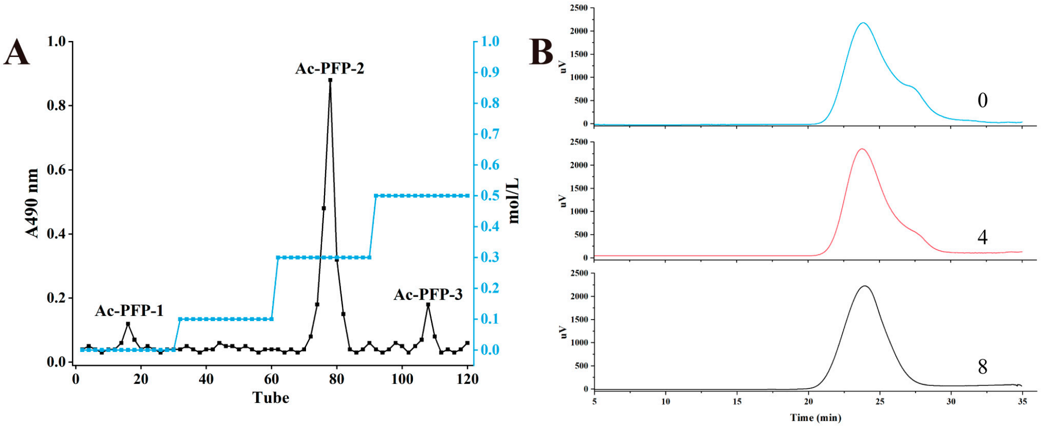

Figure S1 (A) PFP gradient elution curves of DEAE cellulose columns; (B) HPGPC profile during PFP purification

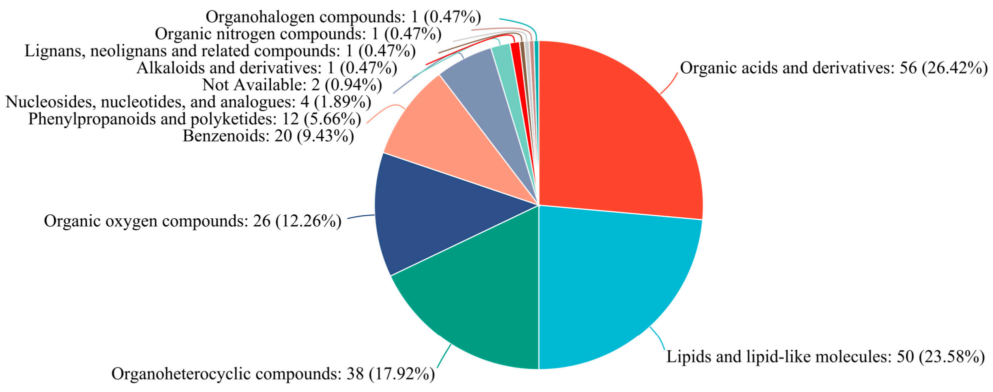

Figure S2 Compound classification of the Human Metabolome Database (HMDB)
